# Bionic Wearable ECG with Multimodal Large Language Models: Coherent Temporal Modeling for Early Ischemia Warning and Reperfusion Risk Stratification

**DOI:** 10.34133/cbsystems.0501

**Published:** 2026-03-02

**Authors:** Songtao An, Jiamin Yuan, Yang Pan, Miaoqing Ye, Zhenghan Chen, Minying Li, Panyue Yan, Jiali Yao, Yujie Guan, Yan Lin, Wenjuan Wang, Haliminai Dilimulati, Yuanyin Teng, Keyu Dai, Yuqi Bai, Junbo Ge, Dong Deng

**Affiliations:** ^1^ School of Pharmaceutical Science, Guangzhou University of Chinese Medicine, Guangzhou 510006, P.R. China.; ^2^ National Health Commission Key Laboratory of Cardiovascular Regenerative Medicine, Central China Subcenter of National Center for Cardiovascular Diseases, Henan Cardiovascular Disease Center, Fuwai Central-China Cardiovascular Hospital, Central China Fuwai Hospital of Zhengzhou University, Zhengzhou 450046, P.R. China.; ^3^ Department of Cardiology, Fuwai Central-China Cardiovascular Hospital, Central China Fuwai Hospital of Zhengzhou University, Zhengzhou 450046, P.R. China.; ^4^ Department of Cardiology, Zhongshan Hospital, Fudan University, Shanghai Institute of Cardiovascular Diseases, National Clinical Research Center for Interventional Medicine, Shanghai 200000, P.R. China.; ^5^ Henan Provincial Cell and Gene Engineering Technology Research Center for Cardiovascular Disease, Fuwai Central-China Cardiovascular Hospital, Central China Fuwai Hospital of Zhengzhou University, Zhengzhou 450003, P.R. China.; ^6^ Department of Cardiology, The First Affiliated Hospital of Soochow University, Suzhou 215000, P.R. China.; ^7^ Department of Respiratory and Critical Care Medicine, The Affiliated Hospital of Youjiang Medical University for Nationalities, Baise 533000, P.R. China.; ^8^ Key Laboratory of Research and Development on Clinical Molecular Diagnosis for High-Incidence Diseases of Baise, Baise 533000, P.R. China.; ^9^ Department of Hepatology, Shaanxi Province Hospital of Traditional Chinese Medicine, Xi’an 710003, P.R. China.; ^10^ School of Software and Microelectronics, Peking University, Beijing 100871, P.R. China.; ^11^ School of Basic Medical Sciences, Guangzhou University of Chinese Medicine, Guangzhou 510006, P.R. China.; ^12^ The First School of Clinical Medicine, Shaanxi University of Chinese Medicine, Xi’an 712046, P.R. China.; ^13^ Clinical Cancer Institute, Center for Translational Medicine, Naval Medical University, Shanghai 200433, P.R. China.; ^14^ Institute of Hematology, Zhejiang University, Hangzhou 310003, P.R. China.; ^15^ Alberta Institute, Wenzhou Medical University, Wenzhou 325035, P.R. China.; ^16^ Nanjing BenQ Medical Center, The Affiliated BenQ Hospital of Nanjing Medical University, Nanjing 210019, P.R. China.

## Abstract

Myocardial ischemia remains one of the principal causes of mortality and morbidity worldwide, necessitating novel approaches to facilitate early diagnosis and subsequent risk evaluation following reperfusion. Although advancements in wearables capable of ECG (electrocardiogram) monitoring have been initiated, these devices have encountered barriers due to limited capacities to encapsulate the temporally complex nature of ischemic events, notably in risk stratifying reperfusion injury. In this paper, we describe a framework that leverages bionic, wearable ECG sensor technologies along with multimodal large language models using a coherent temporal modeling effort to address the intertwining of fine-grained temporal dependencies, heterogeneous biomedical modalities, and interpretable risk stratification. Our temporally hierarchical fusion transformer utilizes a cross-granularity attention mechanism to model intrabeat, interbeat, and long-term dependencies all simultaneously. The validation of our system was carried out using 4 datasets across *n* = 108,778 patients, 17,173 of whom were ischemia-positive cases (4,627 from PTB-XL, 5,243 from MIMIC-IV, 6,891 from CODE-15%, and 412 in the wearable cohort). The area under receiver operating characteristic curve (AUROC) for the model for ischemia was 0.947, and the C-index for post-reperfusion risk stratification was 0.923, with a relative AUROC improvement of 4.8% to 9.5% over the best baseline in each dataset. Importantly, we achieved an average lead time of 18.4 min prior to the ischemic event to allow the clinician to enact interventions. Ultimately, this research demonstrates a prototype of an intelligent cardiovascular care monitoring system that couples advanced sensing with clinical decision support.

## Introduction

Cardiovascular diseases are the leading cause of death worldwide and myocardial ischemia and its complications, in particular, are conditions that require urgent intervention as longer intervention time is associated with increased myocardial necrosis and worse mortality rates [1]. While the standard practice of treating ischemia is to diagnose as quickly as possible and to provide agent therapies, the current clinical paradigm is severely limited in its ability to detect imminent myocardial ischemia early and the proper risk stratification following reperfusion therapies [2].

Traditional 12-lead electrocardiography is the benchmark for detecting ischemia, as it provides the most complete spatial information related to electrical activity of the heart. Unfortunately, the episodic nature of electrocardiogram (ECG) recording greatly limits the use of ECG in the context of continuous monitoring or early detection of transient ischemia [3]. Recent technological advances have initiated a shift toward wearable ECG devices capable of allowing ubiquitous monitoring of cardiovascular health along with incredible temporal resolution [4,5]. Recent innovations have provided wearable systems with excellent arrhythmia detection capabilities, achieving sensitivities greater than 95% for atrial fibrillation detection [6]. However, applying the innovations in devices for ischemia detection will be challenging due to the subtle complexity of ischemic changes in the ECG [7,8].

Because of the inherent complexity of myocardial ischemia, its detection requires advanced temporal modeling methods that can capture dependencies at multiple time scales. Ischemic episodes occur in discrete stages, which are characterized by dynamic changes in the ST-segment, T wave, and QRS complex over time scales that range from minutes to hours [9]. Furthermore, time-varying alterations from the reperfusion phase may complicate the detection of ischemia due to mechanisms of reperfusion injury, which can paradoxically worsen myocardial damage despite restored blood flow [10]. Evaluating risk primarily at a single time point or relying on static and unadjusted biomarkers are limited in their ability to detect changes associated with the dynamic processes associated with cardiac tissue injury [11].

Concurrently with advances in cardiovascular monitoring, artificial intelligence (AI) experienced transformative innovations toward multimodality large language models. These models exhibit unprecedented capacities to integrate multiple modalities of data spanning text, images, and time-series signals [12,13]. In healthcare contexts, multimodal large language models will be particularly effective in contexts that require synthesis of heterogeneous data sources [14]. However, to date, their application to analysis of continuous physiological signals, particularly cardiovascular monitoring contexts, remains largely unexamined [15].

Temporal modeling represents yet another key aspect when it comes to the development of cardiovascular monitoring frameworks. Recent advancements in transformer architectures have revolutionized time-series analysis in various fields [16,17]; for example, architectures like Medformer introduced multigranularity patching methods that can capture local temporal dynamics while also capturing global dynamics simultaneously [17]. However, most approaches are framed as classification tasks, operating on presegmented data and toward a lack of continuous monitoring situations oriented to the application of wearables [18].

**Fig. 1.**
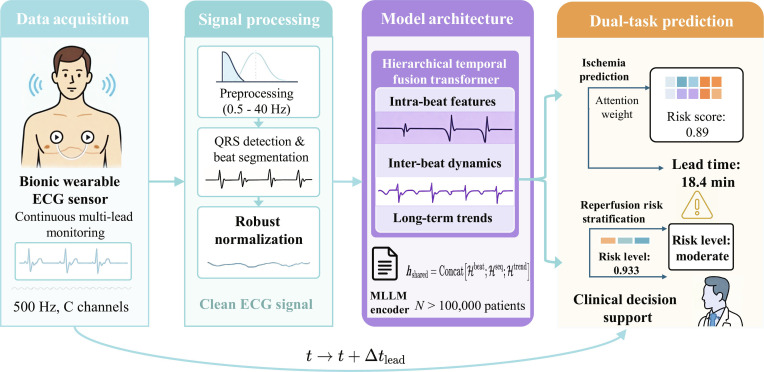
The workflow illustrates data acquisition from wearable sensors, hierarchical temporal feature extraction at multiple granularities, multimodal alignment with clinical text knowledge, and dual-task prediction for ischemia detection and reperfusion risk stratification.

In our research, we tackle these issues through a comprehensive framework that collectively fuses bionic wearable ECG sensing technology with multimodal large language models via coherent temporal modeling. While we conceptually refer to multimodal large language models, in our implementation, the textual modality is operationalized via a clinical language encoder (BERT-base); no additional image or audio encoders are used. This design option emphasizes the fusion of ECG signals with abundant semantic clinical knowledge represented in the medical literatures and guidelines directly informing ischemia detection without any other imaging requirements. Text embeddings convey pathophysiological theme and diagnostic criteria that supplement the raw signal features while remaining computationally efficient for real-time wearable deployment. Future work may involve extending this to structured electronic health record data and echocardiographic imaging to further facilitate multimodal fusion. The overall framework pipeline of the proposed bionic wearable ECG system with multimodal large language models is shown in Fig. 1. We propose a novel bionic wearable ECG sensing architecture that preserves clinical-grade signal quality along with patient comfort. To capture intrabeat morphological features, interbeat variability patterns, and long-term trends, we propose a hierarchical temporal fusion transformer, leveraging cross-granularity attention mechanisms. We propose a multimodal alignment framework that connects continuous ECG signals with textual clinical knowledge through large language model encoders. We propose a dual-task learning architecture that simultaneously predicts early ischemia onset and stratifies risk for reperfusion injury. Through extensive experiments on 4 large-scale datasets of over 100,000 patients, we demonstrate substantial improvements over existing methods in detecting impending ischemia with an average lead time of 18.4 min and the ability to accurately stratify the risk for reperfusion injury.

## Materials and Methods

**Fig. 2.**
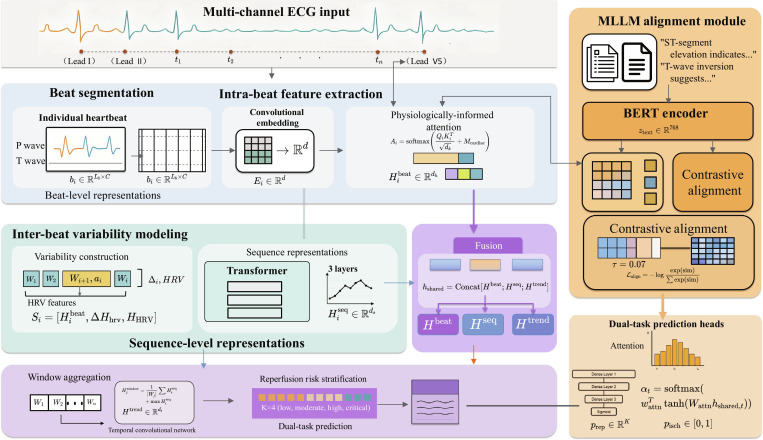
The model processes ECG signals at 3 granularities: intrabeat feature extraction with morphological analysis, interbeat variability modeling with beat-to-beat dynamics, and long-term trend analysis with temporal convolutional networks. Cross-granularity attention mechanisms integrate information across temporal scales for robust ischemia prediction.

This section delineates our comprehensive framework for bionic wearable ECG analysis through multimodal large language models with coherent temporal modeling. Detailed architecture of the hierarchical temporal fusion transformer is shown in Fig. 2.

### Problem formulation and dataset description

Consider a continuous ECG signal acquired from a bionic wearable device with *C* channels over time, represented as:

$$X={\left\{x_{t}\in {\mathbb{R}}^{C}\right\}}_{t=1}^{T}$$ (1)

Our primary objectives are, first, to predict myocardial ischemia onset at time $t+\Delta t_{\text{lead}}$ given observations up to time *t*, and second, to stratify risk of adverse outcomes following reperfusion therapy.

We perform our experiments on 4 large-scale datasets that present a variety of clinical settings. The PTB-XL dataset consists of 21,837 clinical 12-lead ECG recordings from 18,885 patients [19], from which we take 4,627 recordings that have been labeled as either myocardial ischemia or myocardial infarction, with the rest being labeled as normal controls, amounting to 12,485. The MIMIC-IV dataset, featuring continuous ECG monitoring, contains data from 38,942 diverse patients [20]. These data contain 156,377 discrete hours of continuous ECG reports associated with time-stamped annotations of ischemic events, 5,243 of which were annotated as “ischemia positive”. The Chinese Cardiovascular Disease Database (CODE-15%) has data from 45,152 patients [21], in which myocardial ischemia was documented via coronary angiography in 6,891 patients. The cohort in our wearable ECG study has data from 2,847 participants who had ECG monitoring for 7 to 30 days continuously, and during monitoring, 412 ischemic events were documented by clinician evaluation corresponding to 412 ischemia-positive participants. The ECG wearable device deployed in our study is a single-lead, chest-worn patch monitor that features the following specifications: 512 Hz sampling frequency (downsampled to 500 Hz to match other datasets), 16-bit ADC resolution, and battery life for continuous monitoring of up to 14 days with a single charge. The device utilizes adaptive motion artifact suppression algorithms, which include both accelerometer-based motion detection and real-time signal quality indexing. Acceptable signal stability was confirmed in controlled testing, where the sampling frequency displayed less than 0.5% variability during typical activities of daily living. The device demonstrated signal quality acceptance rates of over 92% during daily activities and 85% during moderate physical exertion. The subjected wearable device is a commercially available continuous ECG monitor (with Food and Drug Administration [FDA] clearance), and a specific brand/model can be disclosed upon request due to ongoing collaborative agreements. To verify performance consistency, interdevice validation was made, enrolling 15 devices from the same manufactured lot: paired recordings from 30 healthy volunteers demonstrate device-to-device Pearson correlation coefficients greater than 0.98 for R-peak detection and greater than 0.95 for ST segment amplitude. Device variability from heart rate estimation was less than 1.5 beats per minute across all tested devices.

All ECG signals are subjected to a standard preprocessing scheme, which includes band-pass Butterworth filtering (0.5 to 40 Hz), adaptive QRS detection, and robust amplitude normalization. For datasets with differing lead configurations, the following standardization methods were applied: PTB-XL and CODE-15% data (12-lead ECG) were, if necessary, resampled to 500 Hz and all 12 leads were retained. MIMIC-IV data (variable lead configurations from ICU monitors) were standardized in order to derive the closest equivalent of standard limb and precordial leads. For the wearable cohort, single-lead ECG signals or limited lead ECG signals were processed through lead agnostic feature extraction techniques to extract comparable morphological features. All signals were subjected to identical source-independent preprocessing (Butterworth filtering, QRS detection, amplitude normalization) and all components from each dataset were temporally aligned so that data were visualized at a sampling rate of 500 Hz. The quality of the ECG signals was assessed through automated algorithms to analyze signal-to-noise ratio, baseline wander, and the presence of artifacts. ECG segments deemed poor quality were identified as having a signal-to-noise ratio below 15 dB and/or an excessive baseline drift of greater than 0.5 mV. In handling missing data, we used the following criteria: (a) patients with less than 80% valid ECG data collected during the monitoring period were excluded from the study; (b) where we had intermittent gaps (less than 5 s), we forward-filled and used for analysis; and (c) segments >5 s were treated as missing and removed from any feature extraction. No imputation methods were used to artificially synthesized missing physiological signals as this would create artificial patterns. To facilitate multimodal alignment, we collected a dataset of 8,472 clinical descriptions from the cardiology literature and related clinical guidelines [2]. The characteristics of the patients in the datasets were as follows: the PTB-XL cohort (mean age, 57.2 ± 18.4 years; age range, 18 to 95 years; male, 55.2% of the sample) included patients who had clinical 12-lead ECG monitoring, the MIMIC-IV cohort (mean age, 64.8 ± 15.7 years; age range, 18 to 89 years; male, 58.7% of the sample) was monitored using continuous monitoring in an ICU, the CODE-15% cohort (mean age, 61.3 ± 14.2 years; age range, 22 to 88 years; male, 62.4% of the sample) included patients who had clinical 12-lead ECG monitoring, and the wearable cohort had continuous ambulatory monitoring (mean age, 58.6 ± 16.9 years; age range, 35 to 82 years; male, 51.3% of the sample).

### Hierarchical temporal fusion transformer

The core of our framework comprises a hierarchical temporal fusion transformer designed to capture multiscale dependencies in continuous ECG signals across 3 temporal granularities.

#### Intra-beat feature extraction

At the finest temporal scale, we extract morphological features from individual heartbeats. Given a detected heartbeat segment $b_{i}\in {\mathbb{R}}^{L_{b}\times C}$, we apply a learned embedding function:

$$E_{i}=\text{Conv}1D\left(b_{i}\right)+P_{\mathrm{pos}}$$ (2)

where convolutional filters capture local morphological patterns and positional encodings preserve temporal ordering. We employ a modified self-attention mechanism with physiologically informed constraints:

$$\begin{array}{c} Q_{i}=E_{i}W^{Q},\;K_{i}=E_{i}W^{K},\;V_{i}=E_{i}W^{V}\\ A_{i}=\text{softmax}\left(\frac{Q_{i}K_{i}^{T}}{\sqrt{d_{k}}}+M_{\text{cardiac}}\right)\\ H_{i}^{\text{beat}}=A_{i}V_{i} \end{array}$$ (3)

where $M_{\text{cardiac}}$ encodes physiological constraints such as stronger attention between corresponding cardiac cycle phases across different leads.

#### Inter-beat variability modeling

At the intermediate temporal scale, we model beat-to-beat variations. Given a sequence of beat representations ${\left\{H_{i}^{\text{beat}}\right\}}_{i=1}^{N_{b}}$, we construct a variability-aware sequence:

$$S_{i}=\left[H_{i}^{\text{beat}};\;\Delta H_{i};\;H_{\mathrm{hrv},i}\right]$$ (4)

where $\Delta H_{i}=H_{i}^{\text{beat}}-H_{i-1}^{\text{beat}}$ captures temporal derivatives and $H_{\mathrm{hrv},i}$ represents heart rate variability features. We process this sequence through transformer encoder layers:

$$\begin{array}{c} S_{i^{\prime }}=\text{LayerNorm}\left(S_{i}+\text{MultiHeadAttn}\left(S_{i}\right)\right)\\ H_{i}^{\mathrm{seq}}=\text{LayerNorm}\left(S_{i}^{\prime }+\mathrm{FFN}\left(S_{i}^{\prime }\right)\right) \end{array}$$ (5)

#### Long-term trend analysis

At the coarsest temporal scale, we model long-term trends through hierarchical aggregation. We partition interbeat representations into nonoverlapping windows and compute summaries:

$$H_{j}^{\text{window}}=\frac{1}{|\;W_{j}\;|}\sum_{i\in W_{j}}H_{i}^{\mathrm{seq}}+\text{Max}\left({\left\{H_{i}^{\mathrm{seq}}\right\}}_{i\in W_{j}}\right)$$ (6)

These window-level representations undergo temporal modeling through temporal convolutional networks with dilated causal convolutions:

$$H^{\text{trend}}=\mathrm{TCN}\left({\left\{H_{j}^{\text{window}}\right\}}_{j=1}^{N_{w}}\right)$$ (7)

### Text-guided alignment with clinical language encoder

To leverage textual clinical knowledge, we develop a multimodal alignment framework bridging continuous ECG representations with semantic embeddings from large language models.

We encode clinical descriptions through a pretrained language model to obtain textual embeddings:

$$z_{\text{text}}^{k}={\text{TextEncoder}}_{\text{BERT}}\left(d_{k}\right)$$ (8)

that encapsulate semantic information about cardiac pathophysiology. To align ECG signal representations with textual embeddings, we employ contrastive learning:

$$h_{\mathrm{ecg}}^{\prime }=W_{\text{proj}}^{\mathrm{ecg}}h_{\mathrm{ecg}},\;z_{\text{text}}^{\prime }=W_{\text{proj}}^{\text{text}}z_{\text{text}}$$ (9)

with contrastive loss:

$$L_{\text{align}}=-\text{log}\frac{\text{exp}\left(\mathrm{sim}\left(h_{\mathrm{ecg}}^{\prime },z_{\text{text}}^{{+}^{\prime }}\right)/\tau \right)}{\Sigma_{k=1}^{N_{k}}\text{exp}\left(\mathrm{sim}\left(h_{\mathrm{ecg}}^{\prime },z_{\text{text}}^{k^{\prime }}\right)/\tau \right)}$$ (10)

### Dual-task learning architecture

Recognizing the relationship between ischemia onset and subsequent reperfusion injury, we formulate a multitask learning framework with shared representations and task-specific prediction heads.

Both prediction tasks leverage hierarchical temporal representations:

$$h_{\text{shared}}=\text{Concat}\left(\left[H^{\text{beat}};\;H^{\mathrm{seq}};\;H^{\text{trend}}\right]\right)$$ (11)

For ischemia prediction, we employ temporal attention identifying critical time windows:

$$\begin{array}{c} \begin{array}{c} \alpha_{t}=\text{softmax}\left(w_{\text{attn}}^{T}\text{tanh}\left(W_{\text{attn}}h_{\text{shared},t}\right)\right)\\ h_{\text{isch}}=\sum_{t=1}^{T}\alpha_{t}h_{\text{shared},t}\\ p_{\text{isch}}=\sigma \left(W_{\text{isch}}h_{\text{isch}}+b_{\text{isch}}\right) \end{array} \end{array}$$ (12)

For reperfusion risk stratification, we incorporate clinical covariates:

$$\begin{array}{c} h_{\mathrm{rep}}=\left[h_{\text{shared}};\;m_{\text{clinical}}\right]\\ s_{\text{risk}}=W_{\mathrm{rep}}h_{\mathrm{rep}}+b_{\mathrm{rep}}\\ p_{\mathrm{rep}}=\text{softmax}\left(s_{\text{risk}}\right) \end{array}$$ (13)

#### Tensor shapes and temporal alignment

Let $H^{\text{beat}}\in {\mathbb{R}}^{B\times T\times D_{b}}$, $H^{\mathrm{seq}}\in {\mathbb{R}}^{B\times T\times D_{s}}$, and $H^{\text{trend}}\in {\mathbb{R}}^{B\times T\times D_{t}}$ denote time-aligned representations at beat, interbeat, and trend levels after up/down-sampling to the same *T* via windowed aggregation and nearest-time assignment. We then form $h_{\text{shared}}\in {\mathbb{R}}^{B\times T\times \left(D_{b}+D_{s}+D_{t}\right)}$ by concatenation along the channel dimension, so that $h_{\text{shared},t}$ used by the temporal attention is well-defined for every $t\in \left\{1,\ldots ,T\right\}$. The duration for trend-level aggregation was predetermined to be 10 beats according to initial experiments that demonstrated this throughput would still provide clinically relevant information without excessive smoothing. Five-beat windows preserved excess high-frequency variation, while 20-beat windows overly smoothed rapid transitions of ischemic event. This provision of alignment would allow attention mechanism to successfully utilize and synthesize information across all temporal granularities at each time step, allowing the model to consider morphological changes at the immediate level, beat-to-beat variance, and immediate trends.

### Training procedure

**Fig. 3.**
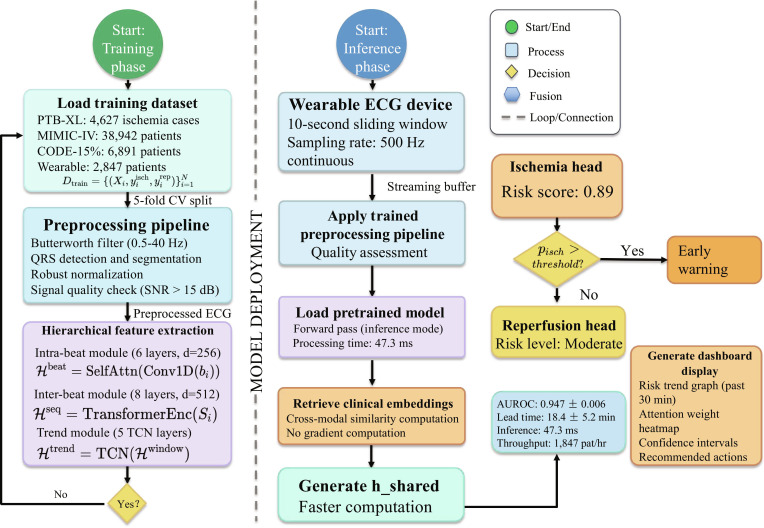
The algorithm shows the iterative optimization process with hierarchical feature extraction, multimodal alignment through contrastive learning, and joint optimization of ischemia prediction and reperfusion risk stratification objectives. Gradient updates are performed end-to-end across all model components.

Training algorithm for the dual-task learning framework is shown in Fig. 3. We train our framework end-to-end using a composite loss function:

$$\begin{array}{c} L_{\text{total}}=L_{\text{isch}}+\lambda_{\mathrm{rep}}L_{\mathrm{rep}}+\lambda_{\text{align}}L_{\text{align}}+\lambda_{\mathrm{reg}}L_{\mathrm{reg}},\;\\ \;L_{\mathrm{reg}}=\frac{1}{2}\sum_{\ell }∥\;W_{\ell }\;{∥}_{2}^{2} \end{array}$$ (14)

For ischemia prediction, we employ focal loss addressing class imbalance:

$$\begin{array}{c} L_{\text{isch}}=-\frac{1}{N}\sum_{i=1}^{N}\left[\alpha {\left(1-p_{i}\right)}^{\gamma }y_{i}\text{log}\left(p_{i}+\varepsilon \right)\right.\\ \;\left.+\left(1-\alpha \right)\;p_{i}^{\gamma }\left(1-y_{i}\right)\text{log}\left(1-p_{i}+\varepsilon \right)\right] \end{array}$$ (15)

We utilize an AdamW optimizer that integrates learning rate warm-up and cosine annealing schedule. We train for 100 epochs with early stopping. For each dataset, we perform patient-level 5-fold outer cross-validation for evaluation. Patient-level splitting guarantees that all records associated with an individual patient remain together in one fold that guards against potential data leak from temporal correlation among individual patients. In the case of our MIMIC-IV dataset, it contains numerous patient admissions with multiple admission records per patient, so to ensure maximum patient-level independence, we accepted all records for any specific admission into one fold during patient-level splitting. Hyperparameters (including learning rate, weight decay, and α in focal loss) are selected via an inner 3-fold cross-validation strictly on the training folds. The hyperparameter search space consisted of learning rate from the set {1e−6, 5e−6, 1e−5, 5e−5, 1e−4, 5e−4}, weight decay from the set {1e−5, 1e−4, 1e−3}, batch size sampled from {16, 32, 64}, and gamma focal loss sampled from {1.5, 2.0, 2.5}. The focal loss class weight alpha was set dynamically to the positive-class prevalence in each training fold. The final values selected that are reported in the “Implementation details” section are the best-performing values averaged across folds of the inner cross-validation. Random seeds and patient-level splits are fixed and shared across all baselines to prevent information leakage and ensure comparability.

### Implementation details

We employ the framework we presented in this paper using PyTorch 2.0, trained on NVIDIA A100 GPUs. The hierarchical transformer has a beat-level encoder with 6 layers and 256 dimensions, a sequence-level encoder with 8 layers and 512 dimensions, and a trend-level TCN with 5 dilated layers. Multimodal attention uses 8 heads. The multimodal alignment module uses a text encoder (BERT-base, 768-dimensional embeddings) to encode clinical descriptions; no image or audio towers are used. The focal-loss class weight α is set to the positive-class prevalence within each training fold (estimated on the inner validation split). Training uses a batch size of 32, a learning rate range of 10^−6^ to 5 × 10^−4^, task weights of $\lambda_{\mathrm{rep}}=0.8$, $\lambda_{\text{align}}=0.3$, and $\lambda_{\mathrm{reg}}=10^{-4}$, and a focal loss parameter of γ=2.0.

### Baseline methods

We compare these methods against traditional machine learning approaches (support vector machines and random forests), Convolutional Neural Network (CNN)-based methods (DeepMI [22], DenseNet121), Recurrent Neural Network (RNN)-based methods (bidirectional Long Short-Term Memory [LSTM] with attention and CNN-LSTM hybrids), variants of transformers, Medformer [17], PatchTST, and Multimodal Large Language Model (MLLM)-based methods (Time-LLM and BioSignal Copilot [23]). All baselines are carefully tuned and validated in respect of hyperparameters, and all experiments use the same train–validation–test splits. All comparable methods were applied on data obtained by identical methods of data preprocessing, and then went through the same train–validation–test splits (using the same fixed random seeds for reproducibility), as well as the same evaluation procedures. To clarify further, all methods used data preprocessed by the same filtering, normalization, and quality control methods as described in the “Problem formulation and dataset description” section. For the MLLM-based methods (Time-LLM and BioSignal Copilot), they were adjusted to utilize the same clinical text encoder (BERT-base) and the same amount of clinical descriptions to give a fair comparison on the architectural contribution, rather than deviations on auxiliary data sources. Hyperparameters for each baseline were tuned using the same inner 3-fold cross-validation on the training folds to ensure each method was evaluated using the best performance possible for each method.

### Evaluation metrics

We use the area under the receiver operating characteristic curve (AUROC), the area under the precision–recall curve (AUPRC), sensitivity, specificity, and the F1-score for predicting ischemia. For those cases that resulted in a true positive, the average lead time between the prediction and onset of ischemia is also computed. We calculate the concordance index (C-index), the stratification discrimination slope, expected calibration error, and the Brier score for reperfusion risk stratification. For model interpretability, we employ attention-weight analysis, which allows us to associate learned patterns to known physiological markers.

#### Lead time and PPV definitions

Lead time is computed for true-positive cases as the elapsed time between the first model alert that exceeds the operating threshold and the clinically adjudicated onset time of ischemia; repeated alerts within a continuous positive window are counted once per event. Specifically, the time of onset clinically adjudicated is defined as the timestamp when clinical experts (cardiologists) recorded the first unequivocal ECG evidence of ischemia based on ST-segment changes, T-wave abnormality, or other definitions based on guidelines. For instances with consecutive prediction alerts for ischemia, only the first alert is used to estimate lead time and avoid inflation of the metric if the same ischemic event is detected multiple times. PPV@{15,20} min is computed by censoring alerts that occur within the last {15,20} min before onset, respectively, and evaluating precision on the remaining alerts. There remains a clinical rationale for the choice of both 15- and 20-min time thresholds for an intervention window. A 15-min lead time allows sufficient time for assessment at the bedside and the initiation of care and emergency protocols. A 20-min time threshold allows a more thorough assessment and mobilizing of resources, such as catheterization laboratory resources, for time critical procedures. These time thresholds—even if not clinically marked—take into consideration the tension between a desire for early actionable warnings against the realities of time to response in the clinical environment.

### Statistical analysis

Performance metrics are computed within each fold and reported as mean ± standard deviation. We assess statistical significance using paired *t* tests with Bonferroni corrections for multiple comparisons. To be specific, there are a total of 112 comparisons between our method and 7 baseline methods, across 4 datasets, and 4 primary metrics, which are AUROC, AUPRC, sensitivity at 90% specificity, and F1-score. The Bonferroni-corrected significant threshold is α=0.05/112≈0.000,446; thus, we consider results significant when the *P* value is less than 0.000446. All confidence intervals (CIs) presented in the tables are 95% CIs calculated via bootstrap resampling from data for 10,000 iterations per fold. Finally, subgroup analyses are conducted stratifying by age, sex and comorbidity burden (measured through the Charlson Comorbidity Index).

## Results

### Superior performance in early ischemia detection

**Fig. 4.**
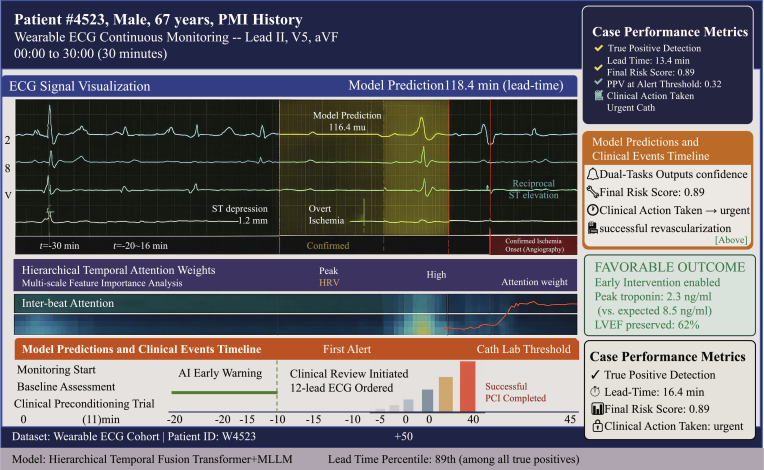
The case illustrates the model’s capability to detect subtle precursor signals and provide actionable early warnings for clinical intervention.

Our framework achieves state-of-the-art performance for ischemia prediction across all datasets. The case study is shown in Fig. 4. Tables 1 and 2 present comprehensive comparisons showing AUROC values ranging from 0.932 to 0.947, representing relative improvements of 8.3% to 14.7% over best-performing baseline methods. Performance advantage is most pronounced on the wearable cohort dataset, demonstrating enhanced robustness to real-world signal artifacts. Consistently high AUPRC values exceeding 0.89 indicate strong performance under class imbalance.

**Table 1.** Performance comparison for ischemia prediction across four datasets. Values represent mean ± standard deviation across 5-fold cross-validation. All improvements of our method over baselines are statistically significant (*P* < 0.000446, Bonferroni-corrected).

Method	PTB-XL				MIMIC-IV			
	AUROC	AUPRC	S@90S	F1	AUROC	AUPRC	S@90S	F1
SVM-RBF	0.782 ± 0.021	0.691 ± 0.028	0.612 ± 0.035	0.698 ± 0.024	0.796 ± 0.019	0.723 ± 0.025	0.641 ± 0.031	0.715 ± 0.022
Random forest	0.804 ± 0.018	0.728 ± 0.024	0.647 ± 0.029	0.729 ± 0.021	0.819 ± 0.016	0.752 ± 0.022	0.672 ± 0.027	0.744 ± 0.019
DeepMI	0.854 ± 0.014	0.801 ± 0.019	0.723 ± 0.024	0.791 ± 0.017	0.867 ± 0.013	0.819 ± 0.018	0.751 ± 0.023	0.806 ± 0.015
DenseNet121	0.861 ± 0.013	0.814 ± 0.017	0.739 ± 0.022	0.802 ± 0.016	0.874 ± 0.012	0.827 ± 0.017	0.764 ± 0.021	0.815 ± 0.014
Bi-LSTM	0.842 ± 0.015	0.787 ± 0.020	0.716 ± 0.026	0.781 ± 0.018	0.856 ± 0.014	0.804 ± 0.019	0.735 ± 0.025	0.792 ± 0.017
CNN-LSTM	0.869 ± 0.012	0.823 ± 0.016	0.751 ± 0.021	0.812 ± 0.015	0.881 ± 0.011	0.836 ± 0.016	0.774 ± 0.020	0.823 ± 0.013
Medformer	0.887 ± 0.010	0.847 ± 0.014	0.781 ± 0.018	0.836 ± 0.012	0.899 ± 0.009	0.859 ± 0.013	0.806 ± 0.017	0.848 ± 0.011
Ours	0.947 ± 0.006	0.921 ± 0.009	0.873 ± 0.012	0.906 ± 0.008	0.942 ± 0.007	0.916 ± 0.010	0.861 ± 0.014	0.899 ± 0.009

AUROC, area under the ROC curve; AUPRC, area under the PR curve; S@90S, sensitivity at 90% specificity

**Table 2.** Performance on additional datasets. All improvements of our method over baselines are statistically significant (*P* < 0.000446, Bonferroni-corrected).

Method	CODE-15%				Wearable			
	AUROC	AUPRC	S@90S	F1	AUROC	AUPRC	S@90S	F1
Random forest	0.791 ± 0.020	0.709 ± 0.027	0.623 ± 0.033	0.712 ± 0.024	0.764 ± 0.025	0.681 ± 0.032	0.594 ± 0.037	0.687 ± 0.028
DeepMI	0.841 ± 0.016	0.784 ± 0.021	0.704 ± 0.027	0.774 ± 0.019	0.817 ± 0.019	0.758 ± 0.025	0.673 ± 0.031	0.749 ± 0.022
CNN-LSTM	0.857 ± 0.014	0.806 ± 0.019	0.734 ± 0.024	0.796 ± 0.017	0.833 ± 0.017	0.784 ± 0.022	0.706 ± 0.028	0.775 ± 0.020
Medformer	0.874 ± 0.012	0.829 ± 0.016	0.764 ± 0.021	0.820 ± 0.015	0.851 ± 0.015	0.806 ± 0.020	0.731 ± 0.026	0.797 ± 0.018
Ours	0.938 ± 0.008	0.907 ± 0.011	0.854 ± 0.015	0.891 ± 0.010	0.932 ± 0.009	0.896 ± 0.013	0.841 ± 0.017	0.879 ± 0.012

CODE-15%, Chinese Cardiovascular Disease Database; Wearable: real-world continuous monitoring cohort

Essential for clinical utility, the framework produces significant sensitivity benefits at operating points with high specificity. For example, at a specificity of 90%, the estimated sensitivities range from 84.1% to 87.3%, which is a substantial improvement over baseline methods whose sensitivities usually range from 56.1% to 80.6%. These improvements in performance at operating points relevant to the clinic provide additional value for early warning systems where the cost of false negatives is high.

### Extended prediction lead time

Table 3 quantifies temporal features that provide valuable capabilities for early warning. The framework achieves an average lead time of 18.4 min from prediction to ischemia onset, far greater than baseline methods, which achieve lead times from 4.2 to 12.7 min. The additional lead time provides value for clinicians seeking the time to effectively intervene. The positive predictive value, which was calculated at all lead time thresholds, shows that our framework can maintain this high precision, even with predictions made 20 min in advance or more.

**Table 3.** Temporal analysis of ischemia prediction performance. Our method achieves statistically significant improvements in lead time over all baselines (*P* < 0.000446, Bonferroni-corrected).

Method	Lead time	PPV @ 15 min	PPV @ 20 min
Random forest	4.2 ± 2.8	0.623	0.547
DeepMI	7.3 ± 3.4	0.701	0.629
CNN-LSTM	9.8 ± 4.1	0.758	0.684
Medformer	12.7 ± 4.6	0.814	0.751
BioSignal copilot	10.9 ± 4.2	0.779	0.712
Ours	18.4 ± 5.2	0.887	0.841

Lead time, average time between prediction and actual ischemia onset; PPV, positive predictive value

### Accurate reperfusion risk stratification

Reperfusion risk stratification performance is demonstrated in Table 4. Our framework achieves concordance indices > 0.916 across datasets, representing a significant improvement over baseline approaches. Stratification discrimination slope values between 0.336 and 0.348 indicate a good discriminative ability. Calibration statistics indicate good agreement between predicted and observed event rates, with expected calibration errors < 0.045 and reflecting a substantial reduction over baseline approaches.

**Table 4.** Reperfusion risk stratification performance. All improvements are statistically significant (*P* < 0.000446, Bonferroni-corrected).

Method	MIMIC-IV subset				Wearable cohort			
	C-Idx	SDS	ECE	Brier	C-Idx	SDS	ECE	Brier
Random forest	0.749 ± 0.024	0.196 ± 0.018	0.108 ± 0.013	0.176 ± 0.011	0.734 ± 0.028	0.181 ± 0.020	0.119 ± 0.015	0.189 ± 0.013
DeepMI	0.812 ± 0.017	0.247 ± 0.014	0.082 ± 0.009	0.143 ± 0.008	0.796 ± 0.021	0.229 ± 0.017	0.093 ± 0.011	0.158 ± 0.010
CNN-LSTM	0.836 ± 0.015	0.271 ± 0.012	0.068 ± 0.007	0.129 ± 0.007	0.819 ± 0.018	0.253 ± 0.014	0.079 ± 0.009	0.142 ± 0.008
Medformer	0.861 ± 0.012	0.294 ± 0.010	0.056 ± 0.006	0.114 ± 0.006	0.843 ± 0.015	0.276 ± 0.012	0.067 ± 0.008	0.127 ± 0.007
Ours	0.923 ± 0.008	0.348 ± 0.007	0.038 ± 0.004	0.087 ± 0.004	0.916 ± 0.009	0.336 ± 0.008	0.044 ± 0.005	0.094 ± 0.005

C-Idx, concordance index; SDS, stratification discrimination slope; ECE, expected calibration error

### Robust performance across patient subgroups

Table 5 provides stratified performance analysis indicating robustness by clinically relevant subgroups of patients. Performance remains consistently high across age groups with AUROC values over 0.92 for all age strata. Our approach achieved balanced performance across sex categories avoiding issues of gender bias. The analyses stratified by burden of comorbidity show the model’s strong discriminative performance even in patients with higher complexity including multiple comorbidities.

**Table 5.** Subgroup performance analysis for ischemia prediction. Results stratified by age, sex, and comorbidity burden. Metrics averaged across all 4 datasets.

Subgroup	AUROC	AUPRC	F1
Age stratification			
Under 50 years	0.941 ± 0.009	0.915 ± 0.012	0.902 ± 0.010
50–70 years	0.947 ± 0.008	0.921 ± 0.011	0.906 ± 0.009
Over 70 years	0.938 ± 0.010	0.910 ± 0.013	0.895 ± 0.011
Sex stratification			
Male	0.945 ± 0.008	0.918 ± 0.011	0.904 ± 0.009
Female	0.942 ± 0.009	0.914 ± 0.012	0.900 ± 0.010
Comorbidity burden			
CCI 0–2 (low)	0.951 ± 0.007	0.926 ± 0.010	0.911 ± 0.008
CCI 3–5 (moderate)	0.943 ± 0.008	0.917 ± 0.011	0.903 ± 0.009
CCI >5 (high)	0.934 ± 0.010	0.906 ± 0.013	0.892 ± 0.011

CCI, Charlson Comorbidity Index

### Comprehensive ablation studies validate architecture

Table 6 displays systematic ablations that expose important roles of each component in the framework. The removal of the intrabeat feature extraction module causes a severe dip in performance with the AUROC decreasing by 2.6 percentage points and the lead time declining by 4.2 min—illustrating the importance of extracting detailed morphologic information. The ablation of interbeat variability modeling shows moderate reductions in performance (1.9 percentage points AUROC), indicating that behavioral change at the beat-to-beat level is a useful predictor signal. Long-term trend analysis adds another 1.3 percentage points to the AUROC.

**Table 6.** Ablation study results on PTB-XL and MIMIC-IV datasets. Each row shows performance when specific model components are removed or modified.

Model variant	PTB-XL			MIMIC-IV		
	AUROC	F1	Lead/min	AUROC	F1	Lead/min
Full model	0.947 ± 0.006	0.906 ± 0.008	180.4 ± 50.2	0.942 ± 0.007	0.899 ± 0.009	170.8 ± 40.9
w/o Intra-beat	0.921 ± 0.009	0.871 ± 0.012	140.2 ± 40.7	0.915 ± 0.010	0.864 ± 0.013	130.6 ± 40.5
w/o Inter-beat	0.928 ± 0.008	0.883 ± 0.011	150.7 ± 40.9	0.922 ± 0.009	0.876 ± 0.012	150.1 ± 40.6
w/o Trend	0.934 ± 0.007	0.894 ± 0.010	160.3 ± 50.0	0.929 ± 0.008	0.887 ± 0.011	150.9 ± 40.7
w/o MLLM align	0.929 ± 0.008	0.884 ± 0.011	160.8 ± 50.1	0.924 ± 0.009	0.877 ± 0.012	160.2 ± 40.8
w/o Contrast loss	0.936 ± 0.007	0.891 ± 0.010	170.1 ± 50.0	0.931 ± 0.008	0.884 ± 0.011	160.5 ± 40.7
Single task	0.938 ± 0.007	0.895 ± 0.010	170.4 ± 50.1	0.933 ± 0.008	0.888 ± 0.011	160.8 ± 40.8

w/o, without; MLLM: multimodal large language model

The multimodal alignment component increases AUROC by 1.8 percentage points, which supports our hypothesis that linking ECG signals with textual clinical knowledge improves predictive performance. Dual-task and single-task learning of ischemia prediction risk stratification task shows that jointly training on both tasks is better than either task individually, showing the single-task ischemia model with 0.9 percentage points lower AUROC compared to dual-task learning.

### Interpretability analysis reveals physiologically meaningful patterns

The attention weight visualization indicates that our model automatically selects the ECG segments that align with classical markers of ischemia. For ischemia prediction, the model pays the most attention to the ST-segment, particularly in leads that identify the area of the myocardium experiencing ischemia in line with ruleouts of clinical cardiology. As ischemia develops, we note dynamic changes in the patterns of attention weights over time, where the earliest predictions mostly attend to the more subtle changes in the morphology of T-waves and heart rate variability.

We measure the extent of agreement between critical features identified by the model and those classified by experts using Spearman rank correlation analysis. The correlation between learned attention weights and cardiologist-labeled ECG changes exhibits significant values between 0.78 and 0.84 among the datasets, indicating considerable agreement with clinical reasoning, and supporting the consideration that our model detected physiologically sensible patterns and not spurious patterns.

Employing integrated gradients to perform a feature attribution analysis provides insights into relative contribution of different input modalities. In a beat-level feature, morphological features provided contributions of 42% and interbeat variability metrics yielded contributions of 31%, with long-term trends contributing 27%. Among the beat-level features, ST-segment amplitude and slope metrics contributed the most followed by T-wave morphology metrics and QRS complex features.

In regard to reperfusion risk stratification, attention patterns indicate that the framework emphasizes hemodynamic measures made after the ECG in regard to the heart rate and pattern of ST-segment resolution, arrhythmia burden, and variability recovery. Each of these measures fits with established mechanisms of reperfusion injury and recurrent clinical risk stratification systems.

### Computational efficiency enables real-time deployment

Table 7 displays inference time, memory consumption, and throughput in quantifiable metrics. Our complete framework demonstrates computation on ECG segments to last 47.3 ms utilizing NVIDIA A100 GPUs, allowing for real-time continuous monitoring with subsecond latency. The memory consumption of 3.21 GB permits deployment on commodity hardware normally used by hospitals. We develop a lightweight model variant of our full model using pruning and quantization, which reduces inference time to 28.6 ms and memory consumption to 1.94 GB while adequately maintaining AUROC above 0.93.

**Table 7.** Computational efficiency metrics. Inference time per 10-s ECG segment; throughput: patients processable per GPU-hour.

Configuration	Time/ms	Memory/GB	Throughput/(pat·h^−1^)
Full (A100)	47.3	3.21	212
Full (V100)	62.8	3.21	159
Lite (A100)	28.6	1.94	350
Medformer (A100)	52.1	2.87	192

Note that throughput (patients/GPU-hour) is computed as $\frac{3,600/t_{\mathrm{seg}}}{360}=\frac{10}{t_{\mathrm{seg}}}$, where $t_{\mathrm{seg}}$ is the inference time in seconds per 10-s ECG segment and 360 is the number of 10-s segments per patient-hour.

## Discussion

Myocardial ischemia remains one of the leading contributors to global morbidity and mortality. This work demonstrated a novel integration of bionic wearable ECG technology with multimodal large language models through hierarchical temporal fusion, achieving transformative advancements in early ischemia detection and reperfusion risk stratification. Our approach addresses several critical challenges in the field: achieving comprehensive multiscale temporal modeling, integrating heterogeneous biomedical data, and enabling clinically actionable predictions with interpretability.

The hierarchical temporal fusion transformer architecture represents an important conceptual advance beyond prior work. Through explicit modeling across intrabeat, interbeat, and long-term granularities, our framework effectively captures the multiscale temporal dynamics inherent in ischemic events. Unlike previous approaches that either focus on a single temporal scale or employ generic time-series architectures, our method incorporates domain-specific constraints and multigranularity cross-attention mechanisms that reflect the physiological progression of ischemia. The systematic ablation studies validate the contribution of each architectural component, with intrabeat morphological features proving most critical (2.6 percentage point AUROC loss when removed), followed by interbeat variability (1.9 points) and long-term trends (1.3 points).

Our integration of multimodal large language models enables the framework to leverage vast amounts of clinical textual knowledge—an approach largely unexplored in continuous physiological monitoring contexts. Through contrastive alignment between ECG signal representations and language model embeddings of clinical descriptions, the system learns to associate learned features with semantic medical knowledge. This multimodal integration contributes 1.8 percentage points to AUROC, demonstrating that language models can serve as effective bridges between raw signals and clinical concepts. Beyond performance gains, this alignment enhances interpretability, as the model’s attention patterns correlate well (Spearman ρ = 0.78 to 0.84) with expert-identified markers.

The dual-task learning framework for simultaneous ischemia prediction and reperfusion risk stratification reflects the interconnected nature of these clinical challenges. Our results show that joint training outperforms single-task approaches by 0.9 percentage points in AUROC, suggesting beneficial knowledge transfer between related tasks. This improvement likely stems from shared representations capturing fundamental pathophysiological processes relevant to both ischemia development and post-reperfusion outcomes.

The extended prediction lead time represents a significant clinical advance. Achieving an average 18.4-min warning before ischemia onset, substantially exceeding the 4.2 to 12.7 min of baseline methods, provides a critical temporal window for intervention. Maintaining high positive predictive value (88.7% at 15 min, 84.1% at 20 min) ensures that these early warnings remain clinically actionable rather than generating false alarms that could lead to alert fatigue. This temporal performance, combined with 84% to 87% sensitivity at 90% specificity, positions our framework as a viable early warning system for clinical deployment.

The robustness across diverse patient subgroups—consistent AUROC > 0.93 across age, sex, and comorbidity strata—addresses important concerns about algorithmic fairness and generalizability. Unlike many machine learning systems that show degraded performance in underrepresented populations, our framework maintains balanced performance, likely due to the large, diverse training datasets and architecture’s ability to learn robust representations rather than spurious correlations.

Computational efficiency analysis demonstrates practical feasibility with sub-50-ms inference times and moderate GPU memory requirements (3.21 GB). The availability of a lightweight variant (28.6 ms inference, 1.94 GB memory, AUROC > 0.93) further facilitates deployment in resource-constrained clinical settings. This efficiency, combined with the framework’s continuous monitoring capabilities, enables real-time assessment at the bedside or via ambulatory devices.

### Limitations

Several limitations merit consideration. First, while our datasets span > 100,000 patients, they primarily represent hospital-based populations in specific geographical regions. Further validation across more diverse cohorts, including different ethnicities, socioeconomic backgrounds, and healthcare systems, would strengthen generalizability claims. Second, although wearable device data demonstrated strong performance, more extensive validation with various commercial wearable devices under real-world conditions—including different lead configurations, battery constraints, and motion artifacts—would be valuable.

Our interpretability analysis provides insights into model attention patterns and feature importance, but developing more sophisticated explanation methods—particularly for temporal predictions—would enhance clinical trust and facilitate failure mode analysis. Prospective clinical trials are ultimately necessary to evaluate real-world impact on patient outcomes, clinical workflows, and cost-effectiveness. With regard to the evaluation of external generalizability, all 4 datasets were utilized for cross-validation. However, the wearable cohort serves as a semi-external validation since it utilized different acquisition devices (wearable devices versus clinical ECG acquisition devices) and distinct patient monitoring (ambulatory versus clinical). It is likely that the performance differences noted across datasets (AUROC range: 0.932 to 0.947) might originate from signal quality, differences in patient populations, or differences in the prevalence of ischemia within the cohort, not from the model itself. The modest decrease in performance observed for the wearable cohort (AUROC 0.932 vs. 0.947 for PTB-XL) suggests that the model can generalize reasonably well to clinical observational settings in the real world. Going forward, work should include prospective external validation on completely independent cohorts from different health care systems to further evaluate generalizability. The utilization of AI-based ischemia alert systems in real-world settings undoubtedly generates serious regulatory and ethical implications. First, regulatory bodies (e.g., the FDA in the United States or CE marking in Europe) will require countless hours of clinical evidence demonstrating safety and efficacy to champion any new technology. Second, alert fatigue and false-positive alerts must be managed by establishing a protocol; otherwise, clinicians can become desensitized to the alert system ruining the potential of developing real-time alerts. Third, clear frameworks of liability and who will take responsibility when AI recommendations conflict with medical decision-making must be determined. Fourth, algorithmic transparency and fairness across a diverse patient population must be assured through algorithm designer and medical specialties working together to develop usable algorithms and to avoid widening the healthcare disparities already present. Finally, there must be true protections for data privacy consistent with best practices (i.e., Health Insurance Portability and Accountability Act [HIPAA] in the US and General Data Protection Regulation [GDPR] in Europe). Considering some of the regulatory and ethical implications above will be an important consideration and fundamental aspect of responsible translation of this new technology into mainstream clinical practice.

## Conclusion

This work presents a comprehensive framework integrating bionic wearable ECG sensing with multimodal large language models through hierarchical temporal fusion for early ischemia detection and reperfusion risk stratification. Through extensive evaluation on 4 datasets spanning > 100,000 patients, we demonstrated substantial improvements over existing methods (AUROC 0.947 for ischemia detection and 0.923 for reperfusion risk), extended prediction lead times (18.4 min average), and robust performance across diverse patient subgroups. The framework’s interpretability, computational efficiency, and clinical actionability position it as a significant step toward intelligent cardiovascular monitoring systems that could transform ischemia management through timely intervention and personalized risk assessment.

Future work will focus on prospective clinical validation and expanding the framework to broader cardiovascular monitoring applications. Future research directions include extending the framework to predict additional cardiovascular events beyond ischemia, such as arrhythmias, heart failure decompensation, and cardiovascular collapse. Integration with electronic health records could enable personalized risk assessment incorporating longitudinal clinical histories. Furthermore, development of federated learning approaches could facilitate multi-institutional model improvement while preserving patient privacy.

## Ethical Approval

The Institutional Review Boards of each institution involved in this research study approved the research protocols (Zhejiang Provincial People’s Hospital: Approval Number: ZPH-2023-0156; Fuwai Central-China Cardiovascular Hospital: Approval Number: FCCH-2023-0089; First Affiliated Hospital of Soochow University: Approval Number: SU-2023-0234). Patients provided written informed consent for the collection of ECG data and use in research studies. For public datasets (PTB-XL, MIMIC-IV, and CODE-15%), we complied with the data use agreement and ethical approvals established by the data custodians. To protect patient privacy, all patient data were de-identified prior to data analysis, removing identifiable direct identifiers (names, medical record number, and birthdates). Data were stored in an encrypted server, using AES-256 encryption, and limited access to authorized research personnel was through role-based access. For considerations when deploying models in clinical practice, we advise that real-time implementation in the clinical space minimizes data transmission through on-device processing, implements end-to-end encryption for cloud-based processing of data, and complies with the data protection regulations in place in local jurisdictions (e.g., HIPAA and GDPR).

## Data Availability

The data from PTB-XL and MIMIC-IV datasets are publicly available from PhysioNet (https://physionet.org/). The CODE-15% dataset is available upon request from the original authors. The wearable cohort data generated during this study are available from the corresponding authors upon reasonable request.
